# Machine learning and deep learning-based approach in smart healthcare: Recent advances, applications, challenges and opportunities

**DOI:** 10.3934/publichealth.2024004

**Published:** 2024-01-05

**Authors:** Anichur Rahman, Tanoy Debnath, Dipanjali Kundu, Md. Saikat Islam Khan, Airin Afroj Aishi, Sadia Sazzad, Mohammad Sayduzzaman, Shahab S. Band

**Affiliations:** 1 Department of CSE, National Institute of Textile Engineering and Research (NITER), Constituent Institute of the University of Dhaka, Savar, Dhaka-1350; 2 Department of CSE, Mawlana Bhashani Science and Technology University, Tangail, Bangladesh; 3 Department of CSE, Green University of Bangladesh, 220/D, Begum Rokeya Sarani, Dhaka -1207, Bangladesh; 4 Department of Computing and Information System, Daffodil International University, Savar, Dhaka, Bangladesh; 5 Department of Information Management, International Graduate School of Artificial Intelligence, National Yunlin University of Science and Technology, Taiwan

**Keywords:** machine learning (ML), deep learning (DL), smart healthcare, internet of things (IoT), feature extraction, data collection, data analysis

## Abstract

In recent years, machine learning (ML) and deep learning (DL) have been the leading approaches to solving various challenges, such as disease predictions, drug discovery, medical image analysis, etc., in intelligent healthcare applications. Further, given the current progress in the fields of ML and DL, there exists the promising potential for both to provide support in the realm of healthcare. This study offered an exhaustive survey on ML and DL for the healthcare system, concentrating on vital state of the art features, integration benefits, applications, prospects and future guidelines. To conduct the research, we found the most prominent journal and conference databases using distinct keywords to discover scholarly consequences. First, we furnished the most current along with cutting-edge progress in ML-DL-based analysis in smart healthcare in a compendious manner. Next, we integrated the advancement of various services for ML and DL, including ML-healthcare, DL-healthcare, and ML-DL-healthcare. We then offered ML and DL-based applications in the healthcare industry. Eventually, we emphasized the research disputes and recommendations for further studies based on our observations.

## 1. Introduction

A new era in healthcare is beginning, one in which extensive medical data will be more and more important. For instance, accuracy in healthcare looks at a range of patient information, in addition to variances within molecular features, Electronic Health Records (EHRs) and surroundings, as well as ways of living. This guarantees that the proper drug is provided to the appropriate patient at the correct point in duration [1], [2]. As a result of increased access to healthcare data, healthcare research has both opportunities and challenges. Discovering the connections between all the data in huge medical datasets is a significant challenge for developing a reliable healthcare system based on data-driven methods, deep learning (DL) and machine learning (ML). In the past, academics have tried to merge data from various sources to create collaborative knowledge sets that may be used for prediction and discovery [3]. DL and ML, technique-based prediction tools, have not been widely deployed in the healthcare industry despite the fact that existing models have significant promise [4], [5]. Biological data's high complexity, variety, dependency on time, sparsity, and unpredictable nature make it difficult to effectively use [6].

Our cities are becoming more digital as a result of vigorous devices equipped with sensors for data collecting, environmental inspection, digital carriage, health betterment, simple entrance to facilities and services, and general assistance requirements for one and all across the urban areas. The term “smart city” is frequently used to describe a digital metropolis. A smart city contains many elements, all connected by increased technology and the requirement to offer its residents high-quality services [7]–[9]. Smart health is one of these elements. The application of cutting-edge technology to improve and provide high-quality healthcare is known as smart health. Therefore, it necessitates the use of astute gadgets, electronic health surveillance equipment, and web services that are entirely associated with a data core in order to draw conclusions about a person's or a community's health. Since many people use smart health, its creation is a great development. This has increased demand for smart health apps in recent years, along with the continued advancement of technology instruments. For instance, with a mobile smartphone and the inbuilt sensors and applications available on these devices, a person may check their blood pressure [10]. Additionally, the effects of weather on health may be explored by the weather or climatic data of a specific location inside a city to learn what sort of regions of the city to escape [11].

It is standard procedure in biomedical research to have a specialist choose the phenotype that will be employed. Nevertheless, supervised feature space definition loses opportunities to find novel patterns and scales poorly. The graphical representations required for the forecasting can instead be automatically found from the raw data using learning-based methodologies. The nonlinear modules known as layers of representation are also used by DL and ML methods to transmute the properties at a specific level (starting over the initial contribution of the data) toward some properties at a more metaphorical level. The DL and ML present a fascinating as well as a contemporary paradigm which is potential for biomedical informatics considering their demonstrated effectiveness in a variety of fields (such as recognition of voices, machine apparition, spontaneous language processing chores, etc.) along with the prompt speed of methodological improvements [12]. The utilization of DL and ML aptitudes in healthcare is currently proposed or is already in use. For example, International Business Machines (IBM) Watson Health uses this technology to interact with patients and healthcare providers and focuses on providing affordable healthcare to a large population; Google DeepMind has put up a strategy to utilize its specialized expertise to enhance healthcare [13], for the purpose of identifying health concerns on computed tomography (CT), scans, Enlitic employs deep neural networks and X-rays [14], and these are just a few examples.

The extensive application of computer-assisted decision-making and outcome evaluation in the provision of healthcare highlights the technical value of modeling expertise and knowledge [15]. On the other hand, conventional rule-based models primarily rely on feature representation of issue domains and are unable to accurately mimic the complexity of human brains. So, we attempt to address this issue by using a deep model. The deep model creates a single model that can imitate human thought processes by fusing feature representation with learning. However, a vast number of medical conditions that could profit from DL and ML approaches haven't been fully investigated. The various advantages of DL and ML, such as improved performance, a lateral learning model with incorporated feature learning, and the aptitude to deal with intricate, diverse information, among others, could be advantageous to the healthcare industry. Additionally, the sparse, noisy, heterogeneous, and time-dependent nature of healthcare data presents a number of challenges for the DL and ML research community [16]. Additionally, better methods and tools are required to integrate DL into clinical decision-support functioning.

In view of this study, we address existing and planned DL and ML applications in medicine, drawing attention to the prominent factors that appreciably influence health care. We don't wish to furnish a comprehensive context on technological particulars (for example, [17], [18]) or common uses of DL and ML [19]. Instead, we emphasize on biological information obtained through clinical imaging, EHRs, and wearable technology. These industries have not yet made considerable use of DL and ML, despite the fact that other data sources, such as the metabolome, antibody one, and other omics data, are projected to be helpful for health monitoring. We consider these aspects in the context of the characteristics and powers of various DL and ML methods, as well as their practical uses in problem-solving [20]. Another key goal of this study is to identify important research opportunities and issues, such as those pertaining to new algorithm design, data-driven hyperparameter learning, model optimization, integrating domain knowledge, and adapting to resource-constrained devices, as well as effective data representation. One possible product of this work is “Future Generation ML-DL Modeling”. Therefore, the purpose of this work is to serve as a reference for scholars and industry professionals who wish to learn about and create data-driven intelligent systems and enhance intelligent healthcare through the use of DL and ML techniques. We have reviewed a significant number of publications with an emphasis on the Preferred Reporting Items for Systematic Reviews and Meta-Analyses (PRISMA) technique, which is a frequently used checklist and guideline for reporting systematic reviews and meta-analyses of studies that evaluate healthcare interventions or other healthcare-related concerns. The following checklist items—justification, eligibility requirements, search method, research selection, data extraction, and synthesis—were covered in this study that coordinated with this technique. The concrete contribution of this paper is summarized as follows:

- We provide a comprehensive outline of ML and DL applications within the healthcare sector.
- We extensively present some areas that can cover the cutting edge based on the key features of ML, DL, and healthcare.
- We efficiently integrate as ML-DL, ML-healthcare, and DL-healthcare based on novel taxonomic strategies that can be able to offer individual services and grouping-based application services.
- We consider some concrete ML, DL and healthcare-based applications from diverse perspectives.
- In addition, we highlight the open issues and challenges and provide a solution path for future research indications.

**Figure 1.**
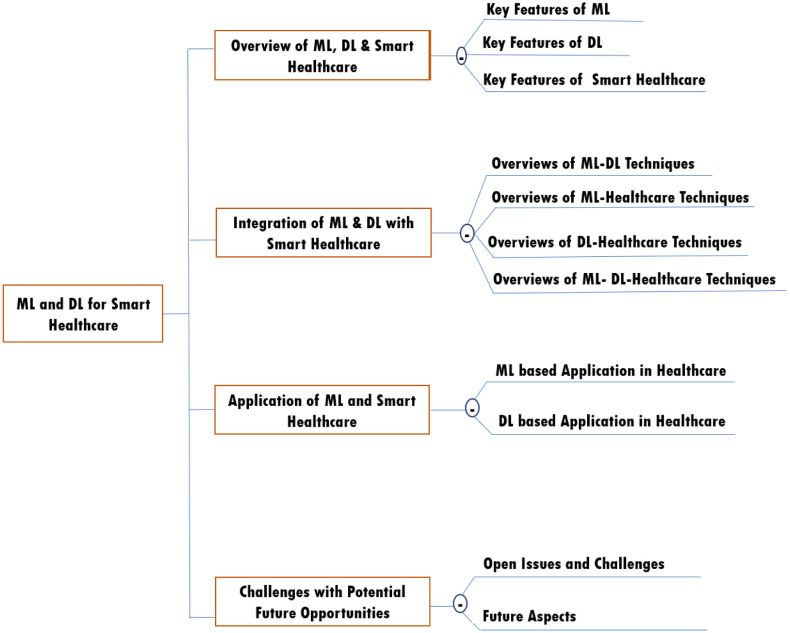
Road-map of this paper.

**Organization of this survey:** This article is arranged as follows: Table 1 displays the abbreviations used in this paper. Section two describes the survey organizing technique PRISMA, which is maintained throughout the research. Section three provides a review of ML, DL and healthcare. After that, section four presents integration among them with a couple of tremendous taxonomies. Applications of ML, DL, and healthcare in diverse areas have been highlighted in Section five. Also, section six considers potential challenges as well as future opportunities in the area of our desire. At last, we conclude this study in Section seven. In summary, this study presents a logical road map in Figure 1.

**Table 1.** List of common abbreviations with description.

**Keys**	**Description**
AI	Artificial Intelligence
ARM	Association Rules Mining
COVID-19	Coronavirus Disease 2019
CNN	Convolutional Neural Network
DoS	Denial of Service
DL	Deep Learning
DT	Decision Tree
HER	Electronic Health Records
FCNN	Fully connected neural network
FL	Federated Learning
GBCAs	Gadolinium-based Contrast Agents
HCU	Healthcare Control Unit
HM	Healthcare Management
HPW	Healthcare Provider's Wallet
IIoT	Industrial Internet of Things
IoMT	Internet of Medical Things
IoT	Internet of Things
kVp	kilo Voltage peak
LR	Logistic Regression
MCC	Matthews Correlation Coefficient
ML	Machine Learning
MHCL	Machine Learning for healthcare communication
RL	Reinforcement learning
XAI	Explainable Artificial Intelligence

## 2. Searching methodology and implementation of PRISMA approach

**Figure 2.**
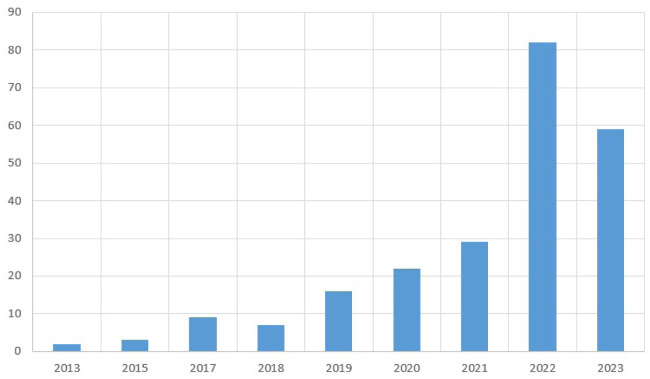
Considered papers mostly cited in this survey.

We have implemented PRISMA in this survey paper. Although there are so many terms and techniques, some of them are considered here that are appropriate for this scenario. The following are the important terminology and principles for this approach:

- A handful of particular phrases have been used in the search of the journals that were part of our analysis.
- For the purpose of accumulating statistics, the majority of recent publications from 2020 to 2023 have been included.
- In Figure 2, a brief synopsis of recent works, such as most of the papers cited from 2022 and second most from 2023, were included in the documentation in this survey.
- The terms “Machine Learning in Healthcare”, “Deep Learning in Healthcare”, and “Machine Learning and Deep Learning in Healthcare” etc. have been used to filter abstracts of various articles and identify pertinent information.
- Certain rules, such as, the articles must be published in a reputable publication with up-to-date content, and several levels of implementation have been maintained.
- A database that was managed during the process has been used to monitor, organize, examine, and incorporate the papers in this survey.

**Figure 3.**
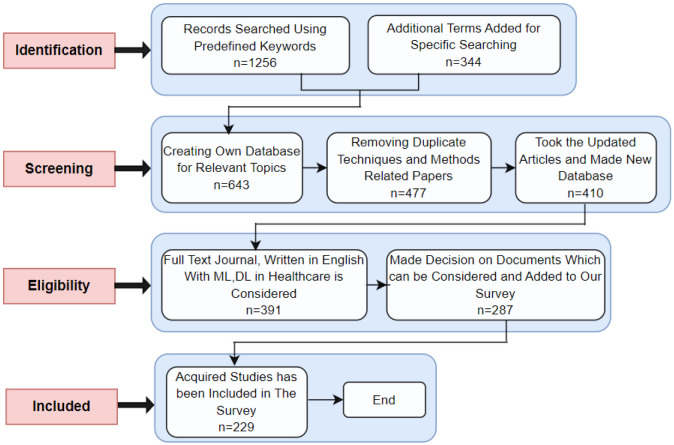
PRISMA-based methodology for this survey.

We have filtered our papers using the above terminology and criteria. In addition, Figure 3 described the step-by-step process used in this study to adopt PRISMA technology. For the identification process, we have searched papers with predefined keywords along with some added specific terms. In the screening part, the duplication of some papers has been removed, and in the eligibility section, the eligible studies have been considered. After finding the required papers, the inclusion part has been completed.

## 3. ML, DL, and smart healthcare: State of the art

The utilization of ML and DL together in the healthcare industry is relatively new and hasn't been fully investigated before. The medical healthcare industry is a promising subject according to current research trends, and we will discuss some of the most important recent literature in the following sections regarding the methods, contributions, and applications of ML-DL to the many fields of this industry. A discussion of the surveys (i.e., 49 papers) about DL and ML technologies, together with the integration of smart healthcare, is presented in Table 2. Each of the groups shown in this table has had its approach and contributions investigated.

### 3.1. Key features of ML

**Classification:** Categorization is a common way that supervised learning issues are represented. The objective is to develop a technique that, given a limited number of already-known subgroups, determines which group a new result belongs to. The training set is fed instances with known subcategories. Each observation may receive more than one label during categorization as opposed to each observation just receiving one. However, the comprehensive taxonomy of the ML approach applied to healthcare is illustrated in Figure 5.

**Clustering:** Clustering, which seeks to organize data in a way that they are more similar to one another than to observations in other groups, is a standard unsupervised learning technique. In contrast to classification, the clustering is determined by the training data rather than being known beforehand. The three primary types of clustering algorithms are as follows– centroid-based based, hierarchical, and density-based [70]. The first two methods are sometimes merged into a single partitional, which is a class. Many of the solutions mentioned in this article use K-means, one of the more well-known algorithms [71].

**Association Rules Mining (ARM):** ARM is the method that is used to unveil latent relationships between different entities using important metrics. It is one of the most common techniques mostly used in data mining and ML fields Group regulations unsupervised learning also includes mining. Support and confidence metrics are the major metrics used to assess association rules.

**Reinforcement Learning (RL):** Another use of ML that draws on trial-and-error learning is RL. Real-world learning is based on how people learn by observing their environment. It helps agents who aim to get the most out of each task overall. RL considers what to do next in order to categorize data based on the advantages the input provides in the short- and long-term. RL and decision-making are frequently combined; for example, this configuration permits human-level performance in a variety of games.

**Table 2.** Related surveys on ML, DL, and smart healthcare. Within each group, the cutting-edge works are chronologically reported and grouped based on the relevant technology.

**Authors**	**Year**	**Relevant Fields**	**Contribution and Techniques**
Karim et al. [21]	2023		An brief overview of the application of ML models for fighting against flood using U-Netriver and FloodGAN techniques.
Mukerji et al. [22]	2023		An investigation on the neuropsychological characteristics of HIV-positive individuals with data-driven approach
Chen et al. [23]	2022		Study of the multiple convergence phenomenon management through machine learning having variable selection.
Halbouni et al. [24]	2022		Intrusion detection techniques are incorporated for leveraging ML models for mitigating the issues in the cybersecurity sector.
Luan et al. [25]	2021		An analysis of ML-based algorithms in the education field analyzing the algorithms, evaluation measures, and validation.
Reboredo et al. [26]	2021		The most recent developments of machine learning techniques such as NB, SVM, RF, and ANN in the pharmaceutical research.
Verbraeken et al. [27]	2020	ML/MLTechnique	Outlines the benefits and drawbacks of distributed machine learning in comparison to traditional (centralized) machine learning, as well as the techniques used to implement distributed machine learning.
Alanazi et al. [28]	2020		SIR-F and SIR models are proposed, implemented, additionally numerical along with mathematical analysis. Moreover, simulation results are presented for smart health care with the help of mathematical and numerical analyses.
Djenouri et al. [29]	2019		Survey and classification of ML applications in smart buildings to implement occupant-centric and energy centric solutions.

Narayan et al. [30]	2023		A brief explanation of techniques using deep learning to recognize human walking styles at a distance. Using the improvement of Human Gait Activity detection.
Abdusalomov et al. [31]	2023		A Deep Learning-based methodology that is used for the Improvement of forests fire detection technique.
Ibrahim et al. [32]	2022		A study on the deep learning-based techniques to classify the fruits using Convolution Neural Network (CNN)
Aqeel et al. [33]	2022	DL/DL Technique	A thorough investigation of DNA-based encryption using neural networks in the medical sector.
Ahmed et al. [34]	2021		A algorithm which is based on deep learning presents and constructs a noninvasive, automated IoT-based system for tracking and detecting patient discomfort.
Zhang et al. [35]	2021		Reviews recommended systems that use deep learning. Proposed a classification approach for grouping and organizing already-published materials.
Altaheri et al. [36]	2021		DL-based classification of MI-EEG studies from the last ten years. Here, CNN was used to classify MI.
Asraf et al. [37]	2020		Deep learning applications using several dimensions for innovative coronavirus control (COVID-19).
Rahman et al. [38]	2020		A distributed deep learning neural network-based COVID-19 management architecture was presented. Utilizes a distributed DL paradigm, in which one and all COVID-19 edge uses its own local DL framework.

Kumar et al. [39]	2023		A secure Blockchain and Deep learning based model for IoT based healthcare observing system including AutoEncoder (DSAE) with Bidirectional Long Short-Term Memory (BiLSTM).
Gnanasankaran et al. [40]	2023		Analysis NLP techniques of the growth of AI-based application of several areas of the medical field especially after the COVID-19 outbreak.
Azadi et al. [41]	2023		Using network data and deep learning involvement analysis, predicting the long-term viability of hospital distribution networks by developing a network DEA (NDEA) model.
Bhat et al. [42]	2023	Healthcare	The Impact and future opportunities of Deep Learning for Medical sector research areas.
Javaid et al. [43]	2022		Machine learning's relevance in healthcare system an analysis of the characteristics, tenets, and potential by applying sophisticated predictive analytics
Abdullah et al. [44]	2022		A Survey of Probabilistic Deep Learning's Solutions and Constraints in Healthcare
Futoma et al. [45]	2020		Machine Learning and the fallacy of adaptability in medical studies
Wiens et al. [46]	2019		A guide to ethical machine learning in the medical field.

Periyasamy et al. [47]	2023		A research employing predictive modelling techniques to examine the effects of Singapore's elderly population on its medical system includes ANOVA and Correlation.
Patil et al. [48]	2023		This study provides a compressed overview of the application of ML models in the medical arena.
McCoy et al. [49]	2022		To investigate explainability's function in the application of machine learning for healthcare, as well as the requirement and importance of this function for the proper and moral deployment of MLHC.
Sabry et al. [50]	2022	ML-Healthcare	This article presents a survey of the many areas of contemporary automated learning development for wearable medical devices. The difficulties that various gadgets with autonomous learning algorithms face are highlighted using GAN.
Zhang et al. [51]	2022		Deep generative models and federated learning are used in this study as methods to enrich datasets for improved model performance. More advanced transformer algorithms are also used to enhance the simulation of clinical language.
Siddique et al. [52]	2021		This work emphasizes how the use of Machine Learning (ML) in medical communication may help people. The COVID-19 health awareness campaign, treatment for cancer, and imaging-related chatbots are included in this.
Chen et al. [53]	2021		The ethical part of the medical sector leveraging machine learning approach here a finite sequence of well-defined instructions and an algorithm is used.
Souri et al. [54]	2020		A novel machine learning-powered surveillance system for medical that uses the IoT to assess the illnesses of students with SVM.

Mageshkumar et al. [55]	2023		Automated Collection Reuse for Cloud-Based Medical System with Neural Machine Learning Enabled Categorization Model
Dhar et al. [56]	2023		Clinical vision assessment with deep learning issues, increasing explicitness and Reliability
Narayan et al. [57]	2023		A strategy based on real-time health data to improve the effectiveness of deep learning models
Jujjavarapu et al. [58]	2023	DL-Healthcare	Combining heterogeneous deep neural networks with patients' both organized and unorganized health data to predict the need for decompression therapy using classical and generalizability evaluation of DL
Buddenkotte et al. [59]	2023		An efficient model for ovarian cancer by leveraging deep learning methods with well-established “no-new-Net” (nnU-Net) framework.
Rajan et al. [60]	2022		An accurate signal prediction and estimate technique based on Deep Neural Networks was utilized in an advanced learning-based smart-monitor and patient monitoring system for Internet of Things-based medical infrastructure.
Jin et al. [61]	2022		Explainable DL model for a healthcare system with the help of data-driven technologies.

Vinod et al. [62]	2023		Analyzed the application of AI and ML based model for restricting the spread of COVID-19 with AI-driven techniques with performance metrics.
Zohuri et al. [63]	2023		Machine learning and deep learning components powered by artificial intelligence for robustness in the fields of medicine, advertising, homeland security, and other areas with the concept of Big Data solutions.
Hassan et al. [64]	2023		Recent trends, services, and consequences of AI and ML in the forecasting of postoperative risks with the help of AI algorithms.
Jenkins et al. [65]	2022		Medical facilities based on the Internet of Things, portable clinical sensory equipment, and machine and deep learning algorithms for COVID-19 patient examination, diagnosis, tracking, along with therapy.
Saravi et al. [66]	2022	ML-DL-Healthcare	Utilizing mixed ML models for decision-making and artificial intelligence-driven prediction modeling in the field of spine surgery using CNN.
Bahrami et al. [67]	2022		An in-depth examination of ML and DL algorithms for single-lead Electrocardiogram sleep disorder screening. The authors focused on deep CN such as VGG16, ZF-Net and AlexNet, Recurrent network, and also hybrid deep NN.
Stone et al. [68]	2022		COVID-19 Remote Patient Tracking Using ML and DL Techniques with sensor networks for the human body and IoT.
Afshar et al. [69]	2021		Datasets from computerized tomographic scans (COVID-CT-MD and COVID-19) useful for ML and DL. In this work, the images were reproduced by using the filtered backpropagation method.

**Figure 4.**
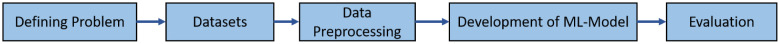
Different stages involved in constructing a learning model.

**Figure 5.**
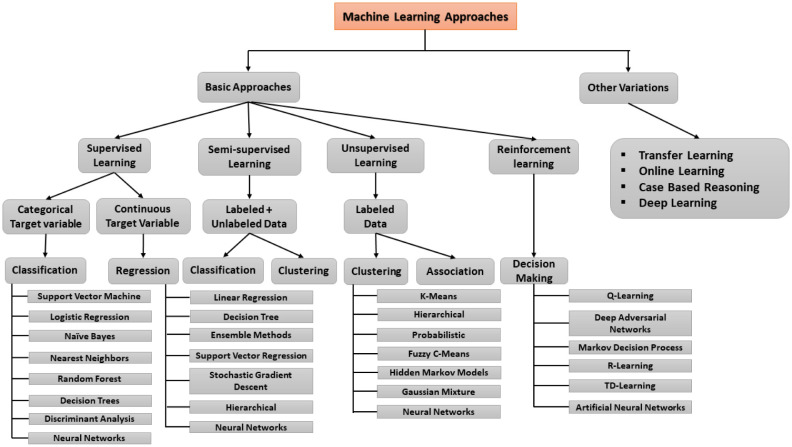
Taxonomies of the ML approach.

#### 3.1.1. Process of ML

Here, we cover every phase of creating a framework for learning in the medical field. Consider that the purpose of this section is to guide scholars toward creating a system of learning for the medical field. Five crucial factors need to be considered while generating a learning model for the healthcare sector: Problem description, information set, preparation of information, ML model production, and assessment. These phases are depicted in Figure 4. Each of these stages is thoroughly explained in the sections that follow.

**Problem Specification:** We must initially consider the following questions while developing an approach to learning for the medical industry: “What is the purpose of designing this learning model?” Identifying healthcare problems and obstacles is the first step in developing a relevant model. The availability of data is an important feature of the first stage. This indicates that data sources should be known to investigators since the information needed to develop and evaluate the learning model should be readily available. A lack of digital data may result in a lack of data in the healthcare sector, patient confidentiality, economic problems, or rare diseases.

**Database:** Datasets are used in industrial healthcare applications to train, validate and test learning models. The healthcare database contains demographic data, pictures, test results, genetic data and sensor data [72]. These data are produced or collected via various platforms, including personal computers, cellphones, mobile applications, network servers, e-health records, genetic data, and wearable technology [73]. Today's global relationships could be enhanced due to the internet and cloud computing [74], [75]. Data accessibility has improved as a result.

**Data Preparation:** When developing a model for the medical sector, data preprocessing is one of the most difficult obstacles to overcome since a ML prototype needs data with greater attributes to deliver better training results and more accurate results. Analyzing disturbance data, absent values, replicate data, and contradicting data is known as data preprocessing. This approach aims to improve the information's integrity before creating the framework for learning. To estimate missing values or filter anomalies, data preprocessing might turn out to be necessary. Furthermore, certain information minimization approaches, such as feature assortment or feature parentage, are perhaps applied if the data has high dimensionality [76], [77].

**ML Model Development:** We must consider the margin of the database, the variety of learning stratagem, and the length of the model inference process while establishing a learning model for the healthcare industry [78]. To prevent overfitting or underfitting, we measure the degree of complexity of a model to learn about the size of the information stored in the database. It's crucial to take a learning model's training duration into account. More parameterized learning models, however, can result in more precise outcomes. Reward-based learning, unsupervised learning, supervised learning, and semi-supervised learning are the four prominent categories of learning techniques [79].

**Evaluation:** Executing numerous procedures to find discrepancies between the system's current behavior and the expected behavior is what evaluation of a ML-based system entails [80]. We must reassess the learning model's performance after being deployed in real situations to assess how it will behave while interacting with actual users [81]. The following components are supposed to be taken on board when assessing the performance as regards to the final model:

- **True Positive (TP):** The percentage of positive class members successfully predicted and classified as positive class members by the classifier.
- **True Negative (TN):** The certain amount of negative category members are adequately prophesied by means of the classifier and recognized as accordingly.
- **False Positive (FP):** The number of unfavorable class members who were wrongly projected as positive class members by the classifier.
- **False Negative (FN):** The number of classes which have positive members who were wrongly anticipated as negative class members by the classifier.

Subsequent that we discuss several relevant degrees for evaluating a learning prototype. This scale is on the basis of the following criteria: TP, TN, FP, and FN:

**Sensitivity:** In order for a classifier to accurately predict a positive outcome whenever the associated ground truth is similarly positive, this scale is denoted as a probability. The true positive rate (TPR), which is another name for this scale, is determined as pursue:

$$\text{Sensitivity}=\frac{TP}{TP+FN}.$$ (1)

**Specificity:** The probability that a classifier will accurately predict a negative outcome when the associated ground truth is likewise a negative result is specified as this scale. The true negative rate (TNR), another name for the specificity, is computed as follows:

$$\text{Specificity}=\frac{TN}{TN+FP}.$$ (2)

**Positive Predicted Value (PPV):** This rating system is characterized as the likelihood that the classification will correctly predict the outcome of the test when the test result (the classifier's output) is positive. PPV is also known as precision, and it is computed as follows:

$$\text{PPV}=\frac{TP}{TP+FP}.$$ (3)

**Negative Predicted Value (NPV):** The probability that a classifier would correctly predict the outcome when the test result is opposite is how this grading system is defined. The computation of this scale appears as follows:

$$\text{NPV}=\frac{TN}{TN+FN}.$$ (4)

**Accuracy:** This scale has a lot of significance. Usually, this scale is used to evaluate classifiers. It is described as the percentage of samples that the classifier is properly categorized. That is determined as follows:

$$\text{Accuracy}=\frac{TP+TN}{TP+TN+FP+FN}.$$ (5)

**Matthews Correlation Coefficient (MCC):** This method is stated as the correlation between the actual results and those projected. Its value ranges from +1 to 1. If MCC=+1, then the classifier correctly anticipates the outcome. If MCC=0, then the classifier cannot prophesy the outcome ameliorate than an arbitrary process. If MCC=1, therefore, there is a complete inconsistency between the expected outcome and the associated basis truth. The MCC margin is determined as follows:

$$MCC=\frac{TP\cdot TN-FP\cdot FN}{\sqrt{\left(TP+FP)\cdot (TP+FN)\cdot (TN+FP)\cdot (TN+FN\right)}}.$$ (6)

**False Discovery Rate (FDR):** The proportion of the total number of favorable outliers to the percentage of positive samples that were mistakenly anticipated is represented by this scale. This is how the FDR scale is created:

$$\text{FDR}=\frac{FP}{FP+TP}.$$ (7)

**Area Under the Receiver Operating Characteristic curve (AU-ROC):** This scale is yet another important variable to consider while assessing classifiers. It is calculated using the area under the ROC curve. It should be noted that the ROC was calculated using TPR and FPR. This scale is derived as follows:

$$AU-ROC=\frac{1}{2}\frac{TP}{TP+FN}+\frac{TN}{TN+FP}.$$ (8)

**F1-Score:** This scale combines the sensitivity and accuracy measures. It is described as the average of their scores, weighted. Recall is a measure of overall amount, whereas precision is a measure of quality. An algorithm with a greater precision will return more relevant results than irrelevant ones, while one with a high recall will return the majority of relevant results, regardless of whether irrelevant results are also returned. We can clarify as follows:

$$Precison=\frac{TP}{TP+FP}\;and\;Recall=\frac{TP}{TP+FN}.$$ (9)

When F1-Score is one, it has reached its ideal value. F1-Score = 0 is said to be the overpower value in contrast. This scale is derived as follows:

$$\text{F1-Score}=2*\frac{Precision*Recall}{Precision+Recall}.$$ (10)

**Figure 6.**
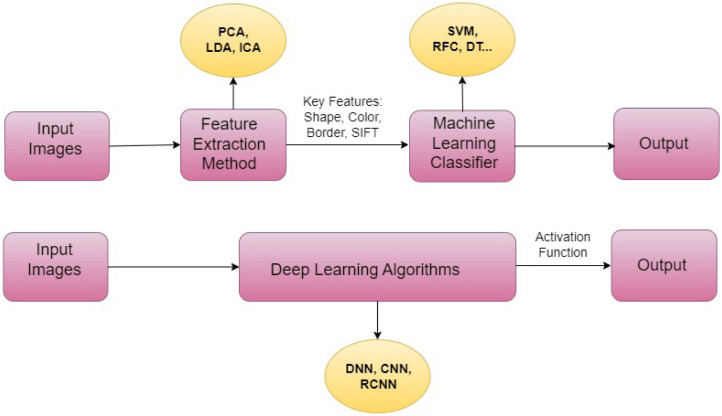
Basic Block Diagram of ML-DL Method.

#### 3.1.2. Role of ML in smart healthcare

**Identification and Diagnosis of Disease:** The primary applications of ML in healthcare are for diagnosing and predicting ailments and conditions that are traditionally seen as being difficult to study. This might include anything from early-stage cancers that are hard to find to other inherited illnesses. The best example of how to combine fast analysis with psychological registration is IBM.

**Engineering and Medical Innovation:** In the new era of medicine production, there are primary clinical learning programs for robots. This also involves research and development (R&D) abilities like next-generation sequencing and precision medicine, which may be used to identify different treatment options for complex illnesses.

**Analysis of Medical Imaging:** Computer visualization is a technical advancement made possible by DL and artificial intelligence (AI). The Microsoft-created Inner Eye activity, which targets picture diagnostic tools for picture analysis, has recognized this. As ML gets more open and as they approach its informative limit, this AI-driven symptomatic interaction turns out to be a component of the objective of grasping new information based on shifting clinical symbols.

**Personalized Medicine:** The combination of prognostic analysis and personal fitness may not truly produce individualized care. Currently, doctors can only do a limited number of studies or assess threats. In the next years, more gadgets and bio-sensors with advanced health assessment capabilities will hit the market, making information easier to obtain for cutting-edge ML-based medical care originalities.

**Integrated Health Records:** The process of maintaining a modern health record is labor-intensive and complicated. The information section measure has been assisted by technology developments, although this is still true for the majority of cycles. In the medical sector, the primary role of ML is to streamline procedures in order to save time, effort, and money. Using vector technologies and ML-based optical character recognition (OCR) recognition algorithms, archives are being organized in an ever-increasing variety of ways.

### 3.2. Key features of DL

**Fully connected neural network:** In a fully connected neural network (FCNN), every neuron in a layer may communicate with every other neuron in the layer above it. For the same reason, these layers are referred to as thick layers. These layers are excessive in terms of computation since each neuron communicates with several other neurons. When there are fewer neurons in the layers, it is preferable to employ this calculation since it would take a lot of time and computing resources to finish the work otherwise. Due to its wide network, it could lead to overfitting [82].

**Convolutional Neural Network (CNN):** CNNs are a particular type of neural network designed to interpret visual input, such as images and sounds. As a result, it is employed in operations such as OCR, object localization, and other picture-processing procedures. CNNs may also be used to recognize text, voice, and video. The pixels that make up an image decide how much white there is in the image. Every pixel in a picture stands for a part that the neural network will look after [83].

**Generative Adversarial Networks (GAN):** Generative adversarial networks (GAN), a single learning process, find and integrate examples from the data as a result. It develops a discriminator- and sub-models-generator-divided model. The generator model aims to create fresh images from the data. With convolutional neural organization, the discriminator model often functions as a parallel classifier. The producer model aims to trick the discriminator model into recognizing the picture, whilst the discriminator model aims to identify fraudulent photos effectively. Both models make every attempt to improve their performance [84].

**The Multilayer Perceptron (MLP):** Between the input and output layers of a feed-forward neural network, known as an MLP, there are frequently one or more hidden layers. In this case, the perceptron can use any activation function and is not constrained to being a strictly binary classifier. As stacked nonlinear transformation layers for learning hierarchical feature representations, MLPs may be thought of in this way.

**Autoencoder:** Unsupervised models called AEs (AEs) attempt to recreate the input data at the output layer. The middle layer, commonly referred to as the bottleneck layer, frequently represents the main characteristics of the incoming data. The denoising AE, sparse AE, variational AE, marginalization AE, and contractive AE are only a few examples of the several types of AEs. However, Figure 8 depicts the comprehensive taxonomy of the DL approach applied to smart healthcare.

#### 3.2.1. DL process

In the case of the DL concept, the extensive aggregation of many neurons between the unseen layers communicates the subsequent propagation concerning the neural network from the activation layer to the end layer. This is made possible by the activation function's nonlinear adjustment. The following is the equation:

$$f(l)=m\left(V^{T}l+s\right).$$ (11)

The activation function is denoted by the letter m in the equation above, where *V^T^* represents a matrix of weights. DL neural network training aims to enhance the neural network's capacity for decision-making and data-suitability [85]. Additionally, the parameters must also be optimized in order to establish the best decision boundary. This requires comparing the loss function, often known as the dissimilarity between the sample's original value, k, and its foretold value, f(l). The equation below can be used to determine the loss function:

c=k−f(l). (12)

The symbol c in the equation above stands for loss. The reduction of the overall loss of all training data is the aim of neural network operation [86]. Feature diagrams can be created considering this layer. The *p*^th^ feature diagram is repeatedly expressed as *m^p^*, and the parameter that makes up the convolution kernel *V^p^* and *s_p_*, then the equivalency below can be acquired:

$$m_{ij}^{p}=f\left({\left(V^{p}*l\right)}_{ij}+s_{p}\right).$$ (13)

The input feature map is represented by one in the previous equation, the activation function is portrayed by f(. ), and the hidden layer parameter is represented by V. Each layer region will be evaluated individually during the convolution computation, and the features will be added to the matrix element multiplication summation and overlaid deviation, as shown below:

$$Z_{h+1}(i,j)=\left[Z_{h}⊗v_{h}\right](i,j)+s=\sum_{a=1}^{K_{h}}\sum_{l=1}^{f}\sum_{k=1}^{f}\left[Z_{p}^{h}\left(b_{0}i+l,b_{0}j+k\right)w_{k}^{h+1}(l,k)\right]+s$$ (14)

$$H_{h+1}=\frac{H_{h}+2k-f}{b_{0}}+1.$$ (15)

In the depicted equations, *s* denotes the degree of deviation, while *Z_h_* and Z_*h*+1_ denote the convolutional input and output of the *h* + 1^th^ layer, which is also known as a feature mapping. The size of *Z_h_*_+1_ is denoted by *H_h_*_+1_, and the measurement of the length and width of the feature are supposed to be the same. The pixels of the related feature map are denoted by *Z*(*i*, *j*), and the amount of channels for the feature map is denoted by *P*. The convolutional layer parameters are *f*, *b*_0_, and *k*, which describe the magnitude of the convolution kernel, convolution stride and padding count. The aforementioned equation can be used to compute the output feature map's sketch if the following conditions are met: The step dimension is B, the filter volume is C, the inlet feature map extent is W, and the amount of zero padding added to the border is K.

(W−C+2K)/B+1 (16)

Similar to how an Artificial Neural Network (ANN) is connected, the completely connected layer is also connected. More variations have been proposed as a result of the CNN network's current rapid development, including the Institute for Global Communications Network (IGCNet) [87], gated CNN (GCNN) [88], GoogleNet [89], AlexNet [90], and the visual geometry group network (VGGNet) [91].

#### 3.2.2. Role of DL in smart healthcare

**Deep Learning in MRI:** Standard magnetic resonance imaging (MRI) is becoming more and more dependent on gadolinium-based contrast agents (GBCAs). Though GBCAs were believed to be benign, they were linked to nephrogenic basic fibrosis, a real, progressive and often severe illness. By using less gadolinium, this testimony may most likely be mitigated. Unexpectedly, low-partition contrast-enhanced MRI frequently produces unsatisfactory demonstration images.

**Figure 7.**
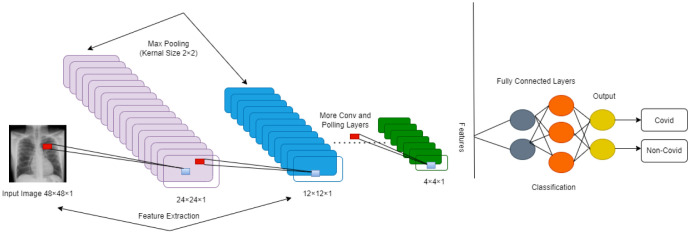
DL process in healthcare.

**Use of DL in CT Scan:** Despite their widespread usage in medical procedures, CT methods pose a radioactive danger. When receiving a physical after more than seven years, for instance, many people would be subjected to 200 chest X-rays worth of radiation, which is the radioactive component of a CT scan. We can see in Figure 7, how the building blocks and layers together are incorporated together to classify a binary decision.

### 3.3. Key features of smart healthcare

- Scanning fingerprints: Mapping of fingerprints.
- Handprint scan: Follows and examines the hand's full pattern.
- Voice recognition: Capturing characteristics and signals of the human voice.

Even yet, there are some crucial procedures that must be done before real-time applications are introduced.

#### 3.3.1. Combining role of ML-DL in smart healthcare

**Finding signs of brain bleeding:** The probability that a patient will recover relies on how quickly a brain hemorrhage is discovered. Analysis shows that these bleeds are understandable anywhere between 12% and 51% of the time and that every year, diseases associated with brain bleeds result in the deaths of almost six million individuals. The level of patient care is greatly reduced by such a vast range of variance.

**Robotic or Robotic-assisted surgery:** In summary, the surgical advancement phase we are currently entering may be described by the integration of surgical robots with AI and data gathered from robotic systems. Motion pursuit data will reveal insights that might fundamentally alter how we now do medical procedures. When the next generation of surgical robots is driven by AI and large information analytics, three of the most promising AI systems—Alpha Go, IBM Watson, and ML algorithms—may be integrated into surgical robots [92].

**Cancer diagnosis:** Malignancies may often be found and identified using ultrasonography, X-rays, MRI, and CT. Unfortunately, certain cancers cannot be accurately recognized enough for these technologies to consistently save lives. Examining microarray sequencing profiles is an alternative technique, although doing so needs several processing hours unless the study is AI-enabled. At this point, it has even been demonstrated that AI-enabled diagnostic algorithmic criteria is as effective at identifying potential skin cancers.

## 4. Integration on ML-DL, ML-healthcare, DL-healthcare, and ML-DL-healthcare: Advancement

### 4.1. Overviews of ML-DL techniques

Whereas DL artificial neural networks can only learn after processing millions of data points, ML versus neural networks is better suited for business scenarios that can collect thousands of data points for the training datasets. Yet, ML maintenance calls for a group of specialists who can manually select features, categorize them, and modify algorithms when they stray [93]. DL involves substantially less human input and is capable of self-correcting when high forecast accuracy is at stake. So, the industry that requires DL has a vast array of unstructured data. In other situations, applying ML algorithms can help you save time [94]. Figure 6 depicts the basic block diagram of the ML-DL method in a healthcare system.

**Figure 8.**
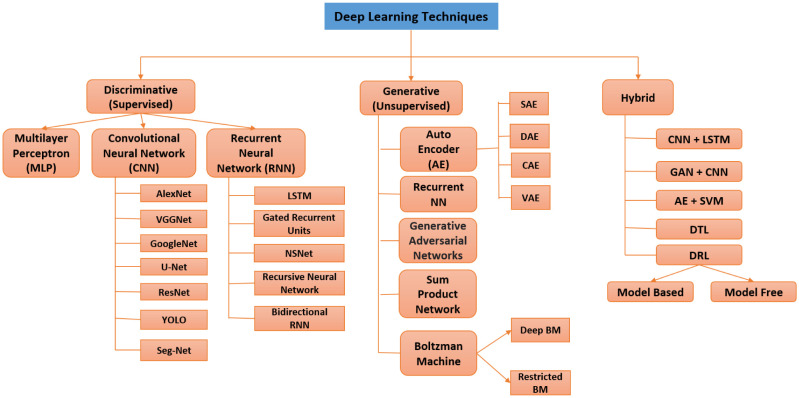
Taxonomies of the DL approach applied to healthcare.

#### 4.1.1. Integration benefits

- Improved customer experience: Customers may stand to gain the most from AI technology. Due to automated chatbots, pushed emails, and other customized communication systems, the time span between client requests and business responses can now be shortened.
- Reducing errors: Once the core of your AI and automation models is in place, you'll notice a reduction in manual errors. Small errors are eliminated because the system only recognizes correctness.
- Automation: It is impossible to discuss speed in relation to DL and ML without bringing up automation. You can allocate resources to ideas and projects that previously appeared impossible by reducing manual processes. Automation may provide businesses more time to focus on strategic planning and the large picture instead of the minute details of individual activities.
- Decision making: Making decisions that are more intelligent has always been the aim of DL and ML together. Humans are capable of critical thought, but we are constrained in how rapidly we can organize and comprehend mountains of data. It has the capacity to transform unprocessed data into an impartial judgment.
- Tackling complex problems: By implementing DL and ML into your business strategy, you can take on more difficult problems. Large-scale solution finding is made possible by these technologies.

### 4.2. Overviews of ML-healthcare techniques

ML techniques are utilized in healthcare to improve patient outcomes by utilizing the expanding amount of health data made available via the IoT. These techniques have a lot of potential applications but also substantial drawbacks. Medical imaging, the natural language processing of medical records, and genetic data are the three principal applications of ML. These fields frequently center on diagnosis, detection, and prediction. By utilizing data and minimizing human engagement, ML is utilized in healthcare to enhance the efficacy and overall character of care. Furthermore, as patient data becomes more available, ML technology will become even more important for healthcare providers and health systems to interpret medical data. In Table 3, the analysis of current works focused on the analysis of smart healthcare and the integration of ML in smart healthcare is shown.

#### 4.2.1. Integration benefits

ML has a spacious assortment of achievable applications in clinical custody, from enhancing patient data, diagnosis and treatment to cutting costs and improving patient safety. Only a few advantages that ML applications in healthcare can offer medical professionals are listed below:

- Improving diagnosis: ML could be applied to the healthcare industry to develop more effective diagnostic systems for looking at medical images. For example, by applying pattern recognition to medical imaging (MRI scans and X-rays), an ML algorithm could be used to find patterns that point to a particular disease.
- Developing new treatments: ML could be used to examine clinical trial data to find previously unidentified drug side effects. This might improve the safety and effectiveness of medical procedures as well as the care given to patients.
- Predictive Approach to Treatment: One example of the significance of ML technology in healthcare is its ability to correctly forecast the development of some of the most fatal conditions in at-risk patients. This addresses oncology, liver and renal diseases, and the detection of diabetes signs (using a Naive Bayes algorithm). [95].
- Data Collection: Medical personnel can choose the most appropriate questions to ask a patient depending on a range of factors by using ML in healthcare management. In addition to forecasting the most likely results, this will make it simpler to gather pertinent information.
- Clinical Research: The process can be sped up with the use of ML algorithms, which is good news. It can be applied to select the best trial sample, gather more data, evaluate trial participants' continuous data, and reduce data-based errors.
- Reducing costs: ML can be used to improve healthcare efficiency, which could lead to lower expenses. For instance, improved devising or patient history supervision algorithms could be evolved in the healthcare industry using ML. This could reduce in contemplation of time and money spent on monotonous chores in the healthcare scheme [96].

### 4.3. Overviews of DL-healthcare techniques

The majority of DL-related news items in the industry at this moment are about diminutive-scale pilots or research schemes that are at present in the pre-commercialization stage. DL, on the other hand, is quickly being applied in creative systems with significant therapeutic applications. Some of the most promising used cases incorporate cutting-edge patient-facing applications as well as a few unexpectedly advanced ways for boosting health information technology (IT) user experiences [97]. Table 4 presents the overview of existing works based on the integration with DL and healthcare with their methods, focused points, and highlighting the drawbacks or challenges of their works.

#### 4.3.1. Integration benefits

- Imaging analytics and diagnostics: CNNs are an example of DL because they are exceptionally good at refinement of images such as X-rays or MRI examine results. The patterns found in actual scans are used to create synthetic CT or MRI images [98].
- Natural language processing: Several of the natural language processing systems that have grown common in the healthcare sector for dictating documentation and converting audio to text are already based on DL and neural networks.
- Drug discovery and precision medicine: Medication development and personalized medicine are other areas of interest for DL developers. Both goals require processing legitimately enormous volumes of genomic, clinical, and population-level data in order to find previously unknown connections between genes, drugs, and physical environment.
- Clinical decision support and predictive analytics: The healthcare territory has great hopes in the field of application along with DL in clinical decision support and predictive analytics for a wide range of disorders. For doctors caring for patients in an inpatient setting, DL may soon prove to be a useful diagnostic tool, warning them of changes in high-risk diseases like sepsis and respiratory failure.

**Figure 9.**
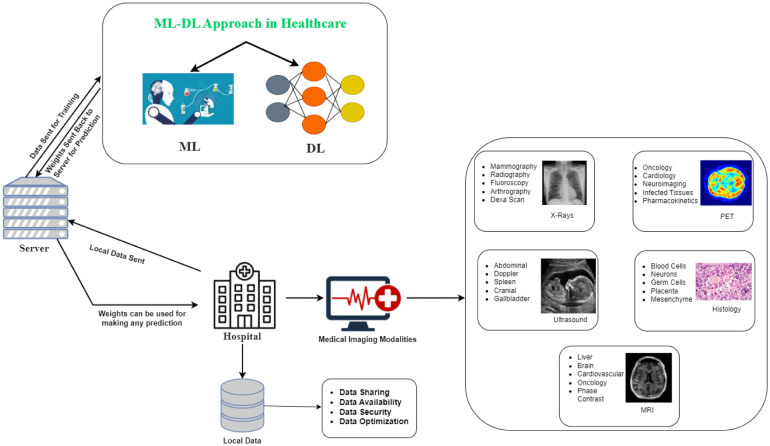
An integration of ML-DL method in healthcare system.

### 4.4. Overviews of ML-DL-healthcare techniques

The current healthcare system places a high value on ML and DL to enhance the treatment provided to patients, doctors, and other healthcare personnel, as well as their overall health. It has been shown to be advantageous to use ML and DL for diagnosing acute illnesses, image scrutiny, drug breakthrough, drug shipment, and smart health surveillance. The effectiveness of previously unavailable treatment options will increase as ML-DL based applications are soon widely implemented and loaded with real-time patient data from a variety of healthcare systems in a variety of nations [99], [100]. Figure 9 illustrates how the ML-DL method has been integrated into the healthcare system.

#### 4.4.1. Integration benefits

DL and ML have made outstanding changes in many areas, including business, industry, schools, colleges, and healthcare systems. As a result of the availability of a wide range of online and offline facilities, we can say that additional changes are being discovered in the medical profession [101], [102]. For the automatic detection of cancer cells, DL is essential. While the DL uses ML to operate independently or autonomously, the ML can address a wide range of issues but calls for human participation. DL, as a contrast to ML, immediately solves the whole problem. Young children, coma patients, and elderly patients can all benefit greatly from DL when it comes to heart disease diagnosis [103].

**Figure 10.**
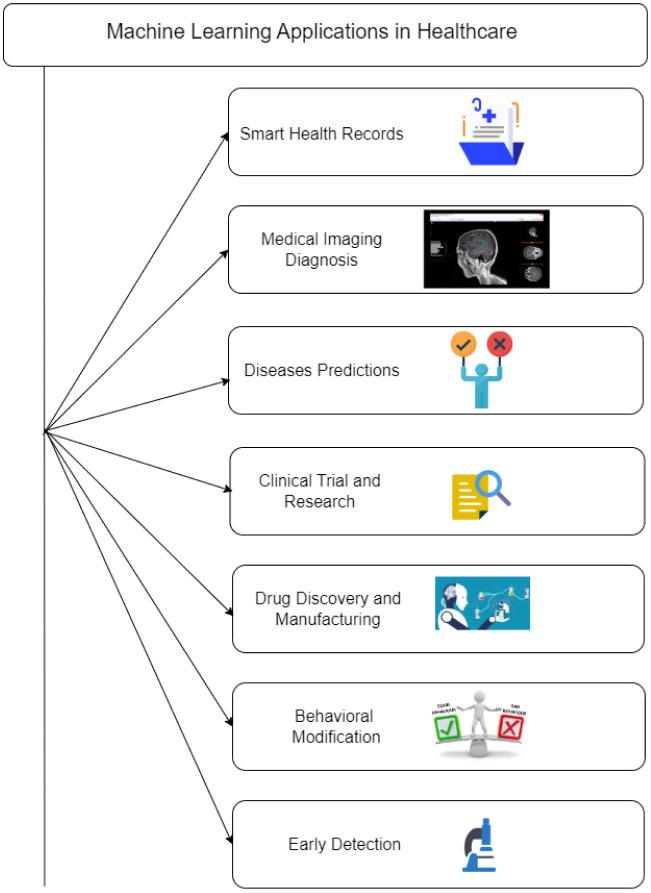
ML applications in healthcare system.

**Table 3.** Analysis of current study based on ML in smart healthcare.

**Works**	**Technologies Used**	**Techniques and Tools**	**Focusing Points**	**Limitations**
Chen et al. [104] (2023)	Information fusion and artificial intelligence	The process of combining many information sources to produce more accurate, consistent, and dependable information to aid in making the best decisions possible is known as information fusion.	Artificial intelligence and information fusion for smart healthcare	Process Under observations.
Chatzinikolaou et al. [105] (2022)	Data Mining and ML	Predictive and descriptive techniques for data mining along with clinical decision support system	Clinical decision support system based on Body area network (BAN) using wearable sensors	Universal interoperation are still not established.
Balakrishnan et al. [106] (2022)	RFID, Wireless sensor and IoT	Radio Frequency Identification (RFID), Wireless Sensor Network, Brainsense headband and smart mobile, all with the Internet of Things (IoT) as its linking platform	To follow the condition of the patient, Smart Healthcare Sensor (SHS) and RFID are used	System based automated prescription can be harmful if the gadgets have a power issue.
Awotunde et al. [107] (2022)	Internet of Medical Things (IoMT) and Artificial intelligence (AI)	Proposed a framework for real-time patient diagnosis and monitoring based on ML and AI-IoMT.	The model was put to the test using a dataset of cytology images, and its performance was assessed based on F-score, accuracy, specificity, sensitivity, and precision	Will seriously reduce human intervention in medical practice.
Bahalul et al. [108] (2022)	IoT, ML and wireless body area network	IoT and ML based security mechanisms as countermeasures to various cyber-attacks	Security mechanism and countermeasures	Smart healthcare in the context of the smart city only.
Singh et al. [109] (2022)	IoT, Federated Learning and blockchain technology	Privacy preservation of IoT healthcare data using Federated Learning and blockchain technology	Privacy preservation of data using the latest technology	Only focuses on Data privacy.
Ahmed et al. [110] (2022)	Explainable Artificial Intelligence	Artificial Intelligence for sustainable smart healthcare	Explainable artificial intelligence in smart healthcare	Sustainable development only.
Verma et al. [111] (2022)	Internet of Things, ML and Artificial Intelligence	Continuous information exchange and physiological data replacement using Internet of Things, ML, and artificial intelligence technologies	Smart Healthcare Cyber-Physical Systems (SHCPS) are the systems of the future that can help the medical community deal with pandemic situations successfully	Universal acceptability and reliability are the main drawbacks.
Rahman et al. [112] (2022)	Federated Learning (FL), Artificial Intelligence (AI), and Explainable Artificial Intelligence (XAI)	The combination of FL, AI, and XAI approaches may be able to reduce a number of systemic constraints and difficulties	The current issues, such as security, privacy, stability, and dependability, may be handled by combining and classifying FL-AI with healthcare technologies	Healthcare is not limited to only AI and FL techniques.
Dwivedi et al. [113] (2022)	IoMT, ML, Previous data	Robots, sensors, telemedicine, remote monitoring, and other related technologies have all assisted in solving a variety of issues with IoMT.	IoMT-based smart gadgets are becoming more and more prevalent, especially in the wake of the worldwide pandemic, and healthcare is no longer solely reliant on these methods	These procedures are not the only ones used in healthcare.
Ghosh et al. [114] (2022)	Statistical and deep learning-based feature analysis with ML	Used facial pain expression databases along with cutting edge techniques during experimentation	Smart sentiment analysis system for pain detection	Medical science is not limited to pain expression only, it needs to address many more fields.
Javaid et al. [43] (2022)	ML and Artificial Intelligence	ML-based techniques assist in detecting early indicators of an epidemic or pandemic	In order to determine if the illness may spiral out of control, the system employs ML to examine satellite data, news and social media reports, and even video sources.	Treatment using previous data is not always enough.
Kondaka et al. [115] (2022)	ML, iCloud Assisted Intensive Deep Learning (iCAIDL)	By bridging between IoT and cloud computing it generates iCAIDL	An intensive healthcare monitoring paradigm by using IoT based ML concepts	Universal operation of this method is not accepted.
Unal et al. [116] (2022)	IoMT, ML and security	Wireless communications, wearable devices, and big data enables continuous supervision of a patient's medical indicators	Continuous monitoring of a patient's medical indicators is made possible by an e-healthcare system, making routine patient follow-ups easier and boosting human productivity	Need to address more medical issues.
Verma et al. [117] (2022)	ML	CNN, Random Forest, Artificial Neural Network (ANN),logistic regression, and Support Vector Machine (SVM)	Review several ML algorithms, applications, techniques, opportunities, and challenges for the healthcare sector	Critical healthcare problem solutions haven't proposed yet.
Kumari et al. [118] (2022)	ML and IoT	Deployment of ML Based Internet of Things Networks for Tele-Medical and Remote Healthcare	This study offers a thorough collection of IoT- and ML-based treatments for patients and telemedicine.	Usable for remote treatment only.
Rehman et al. [119] (2022)	ML, Federated ML and blockchain	Blockchain technology entangled with federated learning technique	RTS-DELM-based secure healthcare 5.0 system	Estimation of intrusion detection.
Kute et al. [120] (2022)	IoT and ML	Application availability, information management, storage, and storage integrity, authentication, trust, and confidentiality	E-healthcare based on the internet of things and ML faces, privacy, security, and trust challenges.	Need to address more scope and solutions.
Talaat et al. [121] (2022)	IoT and ML with EPRAM and Fog computing	Prediction algorithm with fog computing for smart healthcare	EPRAM uses a real-time resource allocation and prediction system to try to manage resources effectively in a foggy environment	Automated prescription systems need to be incorporated.
Swain et al. [122] (2022)	Deep learning, ML and H-ToT	Information collected through healthcare-IoT devices and then DL and ML are applied on them	The majority of the statistical ML (ML) frameworks that are optimized and drive better clinical service delivery are covered in this study.	No measurement of accuracy.
Shakila et al. [123] (2022)	Nature-inspired algorithm, ML	Used Nature-inspired algorithm for feature subset selection and ML for PD classification	Presented a comprehensive analysis of feature selection algorithms and ML models for Parkinson disease diagnosis	Only experimented with ML, DL could be used
Mohanty et al. [124] (2021)	Decision tree, ML, and Random forest	Supervised ML, SVM, Artificial Neural Network, Decision Tree, K-Nearest Neighbor, Random Forest, and Logistic Regression and “Pima Indians (PIDD) dataset”	Utilize historical data analysis to forecast the development of chronic diabetes by handling patients with care.	Universal interoperation is still not established.

**Table 4.** Analysis of current study based on DL in smart healthcare.

**Works**	**Technologies Used**	**Techniques and Tools**	**Focusing Points**	**Drawbacks**
Gupta et al. [125]	Deep learning using edge computing	Deep neural network forecasting for health surveillance with cutting-edge computing	The combination of edge computing and IoT concepts are used in a CNN-based forecasting framework.	Result is shown on specific data.
Kumar et al. [39]	Blockchain, Deep learning, IoMT	Deeper Sparse AutoEncoder (DSAE) combined with Bidirectional Long Short-Term Memory (BiLSTM) is a blockchain-orchestrated deep neural network technique for safe transmission of information in healthcare applications.	A neural networks technique controlled by cryptocurrency for safe data transfer in an IoT-enabled medical facility	Tested for specific dataset only.
Ahmed et al. [126]	IoT, Cloud Computing and Artificial Intelligence	The combination of derived attributes from neural network topologies is accomplished using the concurrent greatest covariance methods, and feature selection is accomplished using a multi-logistic regression controlled entropy variance approach.	To improve the diversity of the knowledge set, data enlargement is performed, while neural network algorithms such as VGG-19 and Inception-V3 are used in conjunction with transfer learning approaches to obtain attributes.	Result is for specific diseases only.
Ravi et al. [127]	Deep learning, Malware detection	Utilizing the portable executable (PE)-Header, PE-Image, PE-Imports, or application programming interface (API) calls, artificial intelligence (AI) is utilized to detect infections.	Hyper-spectral attention-based deep learning framework for malware detection in smart healthcare systems	In intelligent medical facilities, there is a lack of multi-view attention-based deep learning frameworks and powerful feature fusion approaches for recognizing malware.
Jiao et al. [128]	Capsule network, Deep learning, Convolutional neural network	The vital feature extraction ability of neural networks can extract ECG features to solve many problems	The spatial and temporal components of the ECG are extracted using a 1D convolutional neural network (CNN) and a long short-term memory (LSTM) network as an integrated extraction of the features layer.	Accuracy not measured
Ahmed et al. [129]	H-IoT, Deep learning	The technology use the model known as YOLOv3 to detect motion and irritation.	For obtaining the spatial and temporal properties of the ECG, a parallel feature extraction layer comprising long short-term memory (LSTM) network and 1D convolutional neural network (CNN) is implemented.	No measurement of accuracy.
Parida et al. [130]	DL-based detection/classification of diseases	Deep learning based diseases detection and patient monitoring	Deep learning (DL), defeats the limitations of the visual evaluation and the conventional ML	No measurement of accuracy.
Hammad et al. [131]	Deep learning, H-Iot, convolutional neural network (CNN)	Deep Learning Models for Arrhythmia Detection in IoT Healthcare	After representing the input ECG signals in 2D format, the generated pictures are fed into the suggested DLMs for classification.	Efficiency calculated based on noisy data only.
Sujith et al. [132]	Deep Learning, Artificial intelligence	Smart health monitoring using deep learning and Artificial intelligence	By utilizing various IoT, GSM, and SHM modules, DL is beneficial for gathering in-depth information on many important patients, notably those who are coma sufferers.	No measurement of accuracy.
Refaee et al. [133]	Deep learning, Artificial intelligence, IoT	Data are collected from various IoT wearable devices; these data are prepossessed and applied iForest for outlier detection with linear time complexity and high precision	It integrates deep learning, and the internet of things for effective disease diagnosis	Result is outstanding for specific diseases only.
Moqurrab et al. [134]	IoT, cloud computing, Fog computing and Artificial Intelligence	Propose a new model using IoT, cloud computing, Fog computing and Artificial Intelligence	Deep learning is used in a fog-enabled privacy-preserving model to enhance the healthcare system.	Result is for specific diseases only.
Awotunde et al. [135]	Deep learning, IoMT, ArtificialIntelligence	A real-time NF-ToN-IoT dataset for IoT applications that gathered telemetry, operating system, and network data was used to assess the system's performance.	Because IoMT-based devices have a limited capacity for storage and computation, patient health data must be sent to cloud database storage and external computer equipment for processing.	Model attains 89% accuracy over the ToN-IoT dataset only.
Sahu et al. [136]	Deep learning, IoMT, Artificial Intelligence	Constant Verification based on deep learning algorithms for an IoT-enabled medical facility	The system collects data from clients and verifies them using a Deep Learning-based short-term and long-term memory algorithm for categorization that has only been tested on a single dataset.	Tested for specific dataset only
Munnangi et al. [137]	Deep learning, IoMT	Enhanced Deep Learning-Based Approach for Medical Data Analysis in IoT Systems	As a result, the inference of IoT knowledge necessitates the use of effective, lightweight methodologies that are appropriate for this compromise and to validate with limited resources in IoT devices such as wearables.	Result is shown on specific data.

## 5. ML and DL in smart healthcare: Applications

### 5.1. ML-based Applications in Healthcare

The majority of ML applications are employed in the healthcare sector to improve patient outcomes and the level of care, despite the fact that new applications are constantly being developed. You can specialize on ML, which has many applications in the healthcare industry [138]. Figure 10 shows how ML is used in the healthcare sector.

- Disease prediction: ML can be used to find patterns, make connections, and make judgments based on large datasets. Two instances of this are anticipating disease outbreaks in communities and keeping an eye on patient behaviors that worsen health.
- Visualization of biomedical data: ML can be used to create three-dimensional visualizations of biomedical data, including Ribonucleic acid (RNA) sequences, protein structures, and genetic profiles.
- Improved diagnosis and disease identification: Early disease detection will be aided by the discovery of previously unknown symptom patterns and comparison with larger datasets.
- More accurate health records: Improved health record accuracy assures that patients' records are up to date, accurate, and easily accessible for doctors, nurses, and clinic staff.
- AI-assisted surgery: Assist surgeons by doing challenging tasks during surgery, enhancing their comprehension of their work area, and offering procedural completion examples.
- Personalized treatment options: ML may be used to assess multi model data and develop treatment regimens tailored to each patient based on all of the options.
- Medical research and clinical trial improvement: Clinical trial participant selection, data collection techniques, and result analysis can all be enhanced by ML.
- Developing medications: ML can be used to identify new avenues for medication development and to produce innovative therapies that can treat a variety of illnesses.

### 5.2. DL-based applications in healthcare

**Figure 11.**
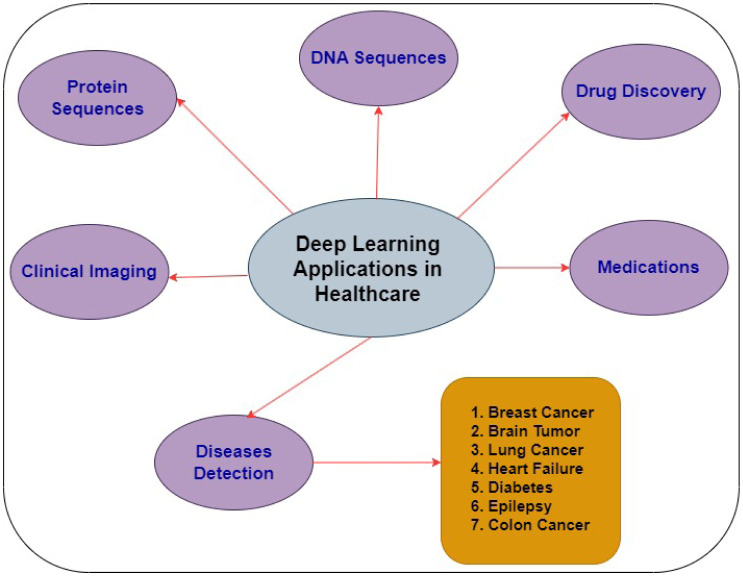
DL applications in healthcare system.

Nowadays, a lot of DL news stories focus on preliminary research projects or small-scale trials. However, DL is gradually making its way into groundbreaking technology with highly beneficial medicinal applications. Moreover, some of the most intriguing use examples involve cutting-edge patient-facing applications and a few surprisingly well-established techniques for improving the user experience of health IT [139]. Figure 11 illustrates the DL applications in the healthcare sector.

- Drug Development: DL helps the pharmaceutical business develop new medicines. The technology looks into the patient's medical background and makes recommendations based on that information. Additionally, this method extracts data from patient tests and symptoms.
- Imaging in Medicine: MRI, CT and Electrocardiogram (ECG)scans are among the medical imaging technologies that can be used to diagnose heart problems, cancer, and brain tumors. DL helps doctors better understand ailments, which enables them to treat patients more effectively.
- Insurance Swindle: Statements involving medical insurance fraud are analyzed using DL. With the use of predictive analytics, it might be able to forecast fraud claims that are anticipated to happen in the future. With the use of thorough education, the insurance business may be able to provide discounts and promotions to its target patient population.
- Alzheimer's Disease: Alzheimer's disease is one of the main issues the medical sector is currently facing. DL is used to power an algorithm for early Alzheimer's disease identification.
- Personalized Medical Treatments: In order to examine people's medical histories, symptoms, and tests, healthcare institutions may use DL algorithms to provide patients with tailored care. Natural Language Processing, or NLP, extracts essential information from free-text medical data for the most common surgical procedures.
- Responding To Patient Queries: Conversational agents that use DL can assist patients or medical staff in identifying trends in a patient's symptoms.
- Audit of Prescriptions: By comparing prescriptions to patient health data, DL algorithms may be able to identify and fix any diagnostic or treatment errors.
- Studying Mental Health: DL models are being used by researchers to enhance mental health clinical practice. Neural networks that are deep are being used in research that seeks to better comprehend the impact of mental illness and other illnesses on the nervous system.

### 5.3. ML-DL-based applications in healthcare

Every problem may be admitted by dint of DL, and performance based on data is constantly superior. Traditional DL can create quick and effective ML for language acquisition while assuring accurate patient data since ML is nonlinear. Patient information or data can be displayed in a variety of forms, including text and images, much like how a patient's status is shown in real time [140]. Real-time results are crucial in IoT applications for healthcare and other industries; therefore, this has been a major problem [141], [142]. DL has the ability to enhance the engineering system for worth control. ML allows for the evaluation of a dynamic system that is capable of changing its environment and recognizing data in various formats. Clinical professionals can diagnose patients' diseases using DL and ML, which can identify normal and abnormal patient data. The basis of DL models is CNN learning, a sort of ANN used for image visualization, including ultrasound, MRI, CT scan and X-ray. In addition to the least, Figure 15 provides a summary of the taxonomy of the ML-DL learning approach used in healthcare applications.

**Figure 12.**
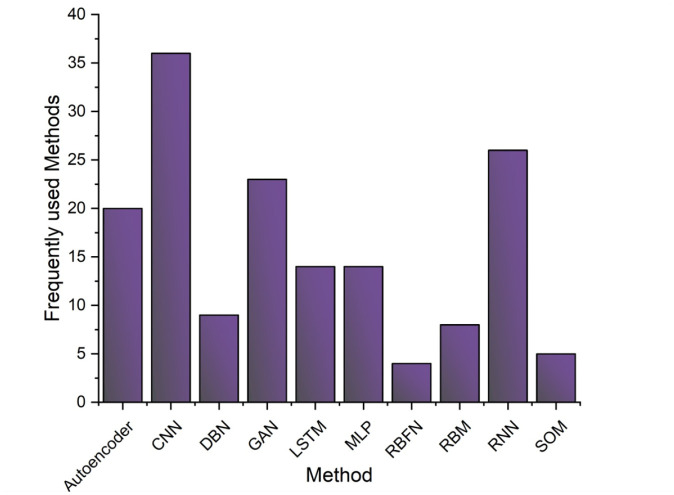
Analysis of commonly utilized methods in the domains of healthcare using ML and DL.

**Figure 13.**
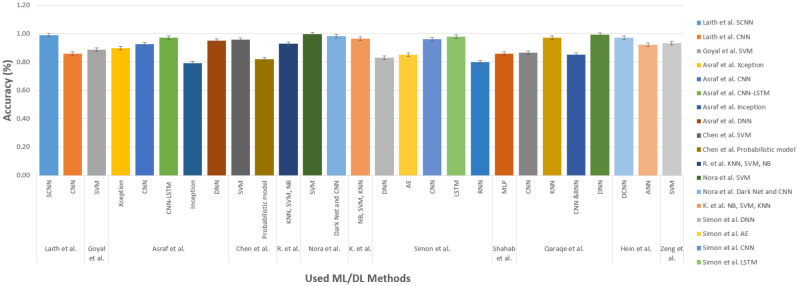
Analysis of the latest studies on various approaches, including their corresponding rates of accuracy.

However, ML-DL supports so many alternative techniques, and they are updating their performance from time to time. In Table 5, there is an overview of various techniques that are used in different case-solving purposes in the vast area of healthcare. Again, Table 6, discusses the datasets that have been utilized by the researchers for the task of solving critical issues in healthcare sectors. In Figure 12, there is a brief analysis of the varieties of techniques mostly used in this field. In addition, according to this figure, the X-axis describes the name of the methods, and the Y-axis explains the frequently used methods. The observation is collected from the Table 5 data along with some others studies from [51], [117][122], [143]–[154]. The CNN model is used for the maximum time. On the other hand, data analysis in ambiguous sectors is a sweltering concern. In Figure 13, a large-scale analysis of the accuracy rate generated from different methods and techniques incorporated with ML-DL in healthcare is presented. Studies from [37], [155]–[164] are plotted in the graph to make an analysis that different methods return different accuracy. The X-axis represents the study in the field of healthcare, and the Y-axis represents the accuracy level found from those studies.

**Table 5.** Overviews of various techniques and Methods used in ML, DL in Healthcare.

References	Techniques of ML-DL										Applications areas specification
	CNNs	LSTMs	RNNs	GANs	RBFNs	MLPs	SOMs	DBNs	RBMs	Autoencoders	
Hamdi Altaheri et al. (2023) [165]	✓	✓	✓	✓	X	✓	X	✓	✓	✓	classification of electroencephalogram
Rasheed et al. (2022) [166]	✓	X	✓	✓	X	✓	X	X	X	X	Providing comprehensive arguments about stabled and trusted areas of ML in healthcare
Kanika Goel et al. (2022) [167]	✓	X	X	X	X	X	X	X	X	✓	Diagnosing COVID-19 in an automated manner
Van der Velden et al. (2022) [168]	✓	✓	✓	X	X	X	X	X	X	✓	Analyzing medical image using explainable artificial intelligence
Matteo Chieregato et al. (2022)[169]	✓	X	X	X	X	X	X	X	X	X	Supporting clinical decision with the help of hybrid model
Messina et al. (2022) [170]	✓	X	X	✓	X	X	✓	X	X	✓	Automatic report generation from medical image
Tharindu Fernando et al. (2021) [164]	✓	✓	✓	✓	X	X	X	✓	X	✓	medial anomaly detection.
Castiglioni et al. (2021)[171]	✓	✓	✓	✓	X	✓	X	X	X	✓	Contrast between ML and DL in medical imaging.
Shahab Shamshirband et al. (2021) [143]	✓	X	✓	X	X	X	X	✓	X	✓	Provide accuracy and applicability of DL model in healthcare solution
Wei Li et al. (2021) [172]	X	X	X	X	X	X	✓	X	X	X	Induced big data analysis in smart healthcare
Laith Alzubaidi et al. (2021) [163]	✓	✓	✓	✓	✓	X	X	✓	✓	✓	Wide range of traditional application of DL
Yakub Kayode Saheed et al. (2021)[173]	X	X	✓	X	X	X	X	X	X	X	Building efficient internet of medical thinks
Irene Y. Chen et al. (2021)[174]	X	X	X	✓	X	X	X	X	X	✓	Probabilistic ML model for Advanced healthcare
Khan Muhammad et al. (2020)[175]	✓	X	X	✓	X	X	X	X	X	X	Brain tumor stage detection for assisting radiologist accurately
Qayyum et al. (2020)[176]	✓	X	✓	✓	X	X	X	X	X	✓	Ensuring a squired state in clinical settings.
Astha Parihar et al. (2020)[177]	✓	X	X	✓	X	X	X	X	✓	X	Examining the current situation of healthcare
akeshi Nakaura et al. (2020) [178]	✓	✓	✓	✓	X	✓	X	X	X	✓	Understanding radiology with ML and DL
Farman Ali et al. (2020) [179]	✓	✓	X	X	X	X	X	✓	X	X	Heart Disease Prediction
Khansa Rasheed et al. (2020) [180]	✓	✓	✓	✓	X	✓	✓	X	X	X	Detection and prevention of Epileptic Seizures
Esteva et al. (2019) [181]	✓	X	✓	X	X	✓	X	X	X	X	Applying computer vision in medical image processing.
Retson et al. (2019) [182]	✓	X	X	X	X	✓	X	X	X	X	Thoracic and Cardiovascular Imaging evaluation
Arwinder Dhillon et al. (2019)[183]	✓	✓	X	X	X	✓	X	✓	X	✓	Analyzing different type of healthcare data
Luca Saba et al. (2019) [184]	✓	X	✓	X	✓	✓	X	✓	X	✓	DL applications in radiology

**Table 6.** Overviews of various datasets utilized in ML, DL, and healthcare.

References	Data Sets Used				Applications Fields
	Discussion of Data Sets	ML	DL	Healthcare	
Huang et al. [185] (2023)	Skin Cancer Classification from ISIC dataset, a public dataset	✓	✓	✓	Classification of skin cancer for assisting dermatologists.
Hu et al. [186] (2023)	Skin Cancer Detection from ISIC 2018 dataset, an open source dataset	✓	✓	✓	A chatbot for detecting seven types of skin cancer.
Soni et al. [187] (2023)	The model was tested using two freely accessible datasets: WISDM and UCI-HAR.	X	✓	✓	Human Activity Recognition Using Deep Learning in Health care.
Kanagala et al. [188] (2023)	Numerous IoT gadgets create massive amounts of info, which is analyzed in order to obtain cognitive data using data analytics.	✓	✓	✓	Efficient digital safety solution for optical information safety for medical applications.
Khan et al. [189] (2023)	Brain tumor Detection from Brats2018, BraTs2019 & BraTs2020, Publicly available dataset	✓	✓	✓	an automated method for identifying brain tumors using three publicly available, unrestricted datasets.
Dua et al. [190] (2023)	Most datasets are collected via IMU, GPS, or ECG while most datasets are used to recognize physical activity or daily activities	X	✓	✓	Human Activity Recognition with Wearable Sensors.
Wang et al. [191] (2023)	In FRESH, physiological data are collected from individuals by wearable devices	X	✓	✓	Architecture for collaborative learning in smart medical facilities while protecting confidentiality.
Baji et al. [192] (2023)	An automated brain tumour identification from the whole brain atlas database	✓	X	✓	To improve brain tumour detection method using k-means clustering and local binary pattern technique.
Hassan et al. [193] (2023)	Cleveland heart disease dataset(open access)	✓	X	✓	For heart disease prediction.
Doshi et al. [194] (2023)	Brain tumor detection from BraTS dataset a publicly available dataset of brain tumour.	✓	X	✓	This approach separates the regions of interest in MRI images to minimize dimensionality.
Hu et al. [195] (2023)	Landsat-BSA datastet for burn patient images (Open source)	✓	X	✓	Burn case treatment.
Ogundepo et al. [196] (2023)	Publicly available Cleveland heart disease dataset	✓	X	✓	Heart disease prediction areas.
Minda et al. [197] (2023)	Medical data	X	✓	✓	Forecasting Algorithm for Multiple Diseases Based on the Finest Deep Learning method.
Raheja et al. [198] (2023)	Cloud-centric data	✓	X	✓	For the diagnosis of cardiac disorders, an IoT-enabled, encrypted clinical health care architecture is used.
Sengar et al. [199] (2023)	RFMiD dataset which is publicly available dataset	✓	X	✓	Assists eye specialists for detection of rental diseases.
Uzun et al. [200] (2023)	Dataset from web scrapping from websites that are publicly available	✓	X	✓	Rapid detection of monkeypox to reduce the spread of the virus.
Jagadeesha et al. [201] (2023)	Fitzpatrick skin type (FST) dataset (open access)	✓	X	✓	Skin tone detection for assisting dermatologists.
Balaha et al. [202] (2023)	HAM10K dataset of Melanoma Classification	✓	X	✓	To aid dermatologists for skin cancer diagnosis from skin images.
Bordoloi et al. [203] (2023)	UCI Dermatology dataset (Public)	✓	X	✓	Skin treatment cases related to skin disorder.
Dileep et al. [204] (2023)	Dataset regarding cardiovascular conditions at UCI (Public) and real-time dataset	✓	X	✓	An automated system for heart disease prediction.
Suha et al. [205] (2022)	An accessible database of patient administrative hospital records from the New York State Department of Healthcare.	✓	X	✓	Patient length of stay forecasting through Random forest model to aid the hospital management system for predicting the proper treatment plan for a patient.
Kundu et al. [206] (2022)	Monkeypox detection from an open source dataset available at kaggle.	✓	✓	✓	a comparison of deep learning and ML methods for monkeypox viral identification.
Rahman et al. [207] (2022)	An accessible database of patient administrative hospital records from the New York State Department of Healthcare.	✓	✓	✓	Predicting the time a patient is hospitalized using a distributed learning approach will help to keep data safe.
Suha et al. [208] (2022)	Kaggle burn patient dataset an open source database.	X	✓	✓	Classification of burn patient images into 3 categories based on severity.

**Table 7.** Existing study analysis of ML-DL with healthcare based on main idea, approaches, and open challenges and further opportunities.

**Authors**	**Main Idea**	**Approaches & Applications**	**Critical Drawback**	**Open Issues and Further Opportunities**
Kundu et al. [206]	Monkeypox detection to restrict the spread of the virus	Vision transformer and conventional ML based approaches.	Insufficient Dataset of the monkeypox disease	To overcome the challenge of the shortage of data GAN can be applied for producing simulated data.
Rahman et al. [207]	Patient Length of stay (LOS) Prediction for assisting healthcare professionals in finding a proper treatment plan.	Federated learning with a linear regression model.	Only linear models have been used.	The model can be tested for other datasets as well.
Solanki et al. [209]	Development of virtual assistant for healthcare sector	Machine earning based chatbot for medical care system.	To gain patients' trust	Provide the ethical part of the system to gain trust of users.
Chen et al. [210]	Leveraging DL for the detection of pancreatic cancer without human intervention	Convolutional neural netwrok with integrated CAD based methods.	A CT scan of the abdomen misses around forty percent of pancreatic cancers that are less than 2 cm.	Collection of real-time data is a challenging task for many countries.
Siar et al. [211]	Brain tumour detection through deep learning model.	CNN, and as a classifier softmax classifier has been used.	Data collection as it may include sensitive information	As it includes healthcare data it requires the security measures needs further consideration.
Awotunde et al. [212]	Breast cancer detection.	Hybrid rule-driven decision-making technique to find five significant insights.	Patients' data privacy is not ensured.	To collaborate with a large number of patient data from patient all around the world.
Özdil et al. [213]	Fatty liver classification	CNN with texture-based feature selection method	The investigation of feature selection using texture is less thorough.	Pre-processing task of thermal images needs further improvements.
Qadri et al. [214]	An automated spine segmentation method through deep learning model	Steps involves preprocessing, regression with sigmoid post processing	To identify fractured vertebrae.	Model needs further improvement to identify some cases for example fractured vertebrae.
Chieregato et al. [169]	Using CNN and CatBoost classifiers, a model that can assist clinical decision-making completely combines imaging and non-imaging data on top of this networking approach.	CNN and catBoost classifier for clinical decision-making tasks.	Clinical decision-making depends on data, but collecting datasets of medical is difficult.	Security needs further discussion, especially for healthcare-based systems.
Saheed et al. [173]	Using DRNN and SML models, an efficient and effective IDS for classifying and foreseeing unexpected cyberattacks in the IoMT environment was presented.	DRNN and SML for cyberattack detection in the IoMT area.	Test within the real-time environment is critical	Further study for Testing within a real-time scenario.
Astha Parihar, Shweta Sharma [215]	using unlabeled data and substantial learning throughout the preparation. AI examines how the genomic landscape interacts across characteristics, whereas DL, MRI, and CT scans are used for illness identification and diagnosis.	AI-based methods for disease diagnosis.	Integration of AI in the healthcare sector requires a vast dataset.	Explainability of such a model helps better comprehend the prediction model's result.
Bhardwaj et al. [216]	Explainable deep learning model for valvular heart disease classification	Deep learning visualization method, CNN architecture for heart disease detection.	Model explainability needs to get the trust of the end user.	Proper training sessions to make the model trustworthy for the end user.
Tasin et al. [217]	A model for diabetes prediction.	SHAP and LIME for model explainability and SMOTE analysis for restricting the class minimization in the area of heart disease detection	dataset contains only 203 samples which might be a critical drawback for model explainability.	Integration of different hospital datasets for diabetes tests may improve the data quality.
Tasnim et al. [218]	A explainable model for mortality prediction	DL methods with integrated SHAP model for explainability of the decision provided by the model for prediction of mortality rate for heart failure based cases.	Security analysis of the model is challenging.	To make the model available for rural areas, the explainability of the model needs further improvement.
Nancy et al. [219]	Smart healthcare monitoring especially for heart disease cases.	IoT with integrated Deep learning model for heart disease prediction	IoT devices memory management is difficult as the devices have limited memory spaces.	Methods that can erase the data saved on the device or a method that does not require saving data, such as Federated learning.

## 6. ML and DL in smart healthcare: Challenges and opportunities

### 6.1. Open issues and challenges

To build a solid model and address some significant medical issues, ML or DL approaches for the healthcare system need real-time datasets. However, gathering data is a challenging endeavour, particularly for the medical system, as it may include patient parties or occasionally hospital authority private information. As a result, there is less interest in data sharing from both patients and hospital staff. The introduction of federated learning [220], [221], which eliminates the need for data transfers from the source, or the use of Blockchain-based security measures for secure and transparent data transfers from source to destination are two privacy measures that need to be focused on in order to deal with this issue [222], [223].

**Figure 14.**
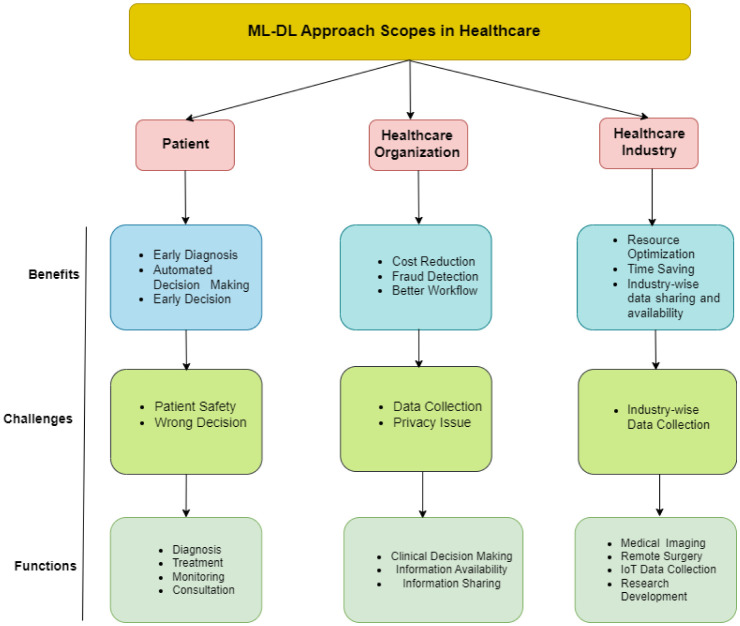
Scopes and challenges of ML-DL method in healthcare system.

The weight of the model is yet another issue with DL deployment. DL produces a heavy model since it uses numerous layers. This substantial model integration becomes extremely challenging in other situations, such as those involving the IoT and wireless sensor networks [224]. To circumvent this difficulty, a lightweight DL model that can perform just as well as typical DL models but is less physically demanding must be used, such as Vision Transformer [225], [226]. Advanced ML or DL models, on the other hand, need a substantial amount of real-time data. However, gathering healthcare data is a difficult task. Thanks to cutting-edge AI techniques that offer data augmentation methods [227], [228]. This augmentation methodology, such as the GAN or time series GAN (ts-gan) [229] method, enables the generation of synthetic data from the already-existing real-time data. In this situation, CNN might be improved in some way and evaluated. Before using the models in the field, a cost analysis for them should be conducted using specific statistical techniques. Figure 14 displays an overview of the use-case sectors and challenges of the ML-DL method in the healthcare system and Table 7 shows existing research in the field of machine and DL technology and its associated research gaps and future directions in this research field.

**Figure 15.**
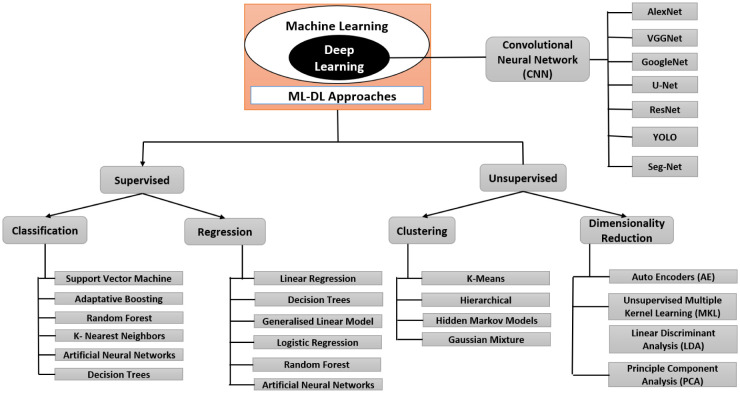
Taxonomies of the ML-DL learning approach applied to healthcare applications.

### 6.2. Overall discussion and limitations

#### 6.2.1. Overall discussion

The aforementioned study aids in understanding how DL and ML automate a number of crucial elements and procedures that medical infrastructure depends on and cannot function without. For example, we find that AI can find every possible way to predict the root cause of an incurable disease and find a related antigen to dissolve it, thereby mitigating its effect; that NLP can automate hospital management processes while saving a significant amount of time and resources; that DL could speed up the diagnosis process. Applications of DL and ML solutions span a range of healthcare domains and tackle contemporary problems in their respective fields of study. Diagnosing different frequent diseases and ailments is one such subfield that adds to the annual rise in the worldwide death rate. A significant portion of this issue can be resolved by utilizing AI-based solutions, particularly DL. Doctors and other workers would also find it challenging to do their work as efficiently at this high pace of population growth due to overwork and a lack of free time. Hospitals can therefore use ML and DL to assist them in overcoming this issue. These technologies can learn to predict every conceivable outcome at every stage with the least amount of error, allowing for the acceleration of further processes while saving crucial time and resources. Using a virtual assistant would be cost-effective, even for hospitals. Thus, it is possible to adopt DL and ML-based systems for diagnosis without a significant financial investment.

Though there are certain restrictions on the use of such cutting-edge technology that would be in charge of making crucial decisions pertaining to people's lives, there might be some difficulties in replacing all of the antiquated or conventionally used technical methodologies that are being used in the current healthcare infrastructure. These problems won't be resolved until every component of the current infrastructure—including the medical staff—supports enhancing the circumstances and the available resources. DL and machine learning-based technologies have the potential to solve many of the most pressing issues in healthcare, and their advancements open up a wide range of interesting and intriguing medical applications that could raise the standard and effectiveness of healthcare delivery. Nevertheless, much study in this area is still needed to fully realize the technology's promise. Moreover, one of the main challenges and obstacles to achieving it is the requirement for a sizable dataset.

#### 6.2.2. Limitations

We draw attention to the following limitations that we believe will be encouraging for DL in healthcare in the future.

- Feature Enrichment: Since there are only so many patients in the world, we should make every effort to learn as much as we can about them in order to better comprehend them and come up with creative solutions for managing them all at once. To develop those qualities, information from EHRs, social media, wearable technology, environment, surveys, online communities, genetic profiles, omics data (including proteomes), and other sources must be used. A crucial and challenging research question would be how to successfully use such incredibly diverse data and incorporate it into a DL model.
- Federated Inference: Every clinical facility has a unique patient base. In this context, developing a DL model that uses patients from several sites while protecting their private information becomes a critical challenge. Consequently, another key area of research that will interact with other mathematical fields like cryptography is how to securely learn deep models in this federated situation.
- Model Privacy: Privacy is a key consideration when growing the usage of DL (using cloud computing services, for example). DL models are much harder to maintain privately since there are more parameters to secure however, some recent efforts have made considerable advancements in this field. Nonetheless, given the amount of private information that deep models are anticipated to handle in clinical applications, the implementation of intelligent technologies for next-generation healthcare has to evaluate these dangers and strive toward establishing a differential privacy norm.
- Including Expert knowledge: For medical issues, the utilization of current expert information is quite beneficial. Research on the incorporation of expert knowledge into DL is essential to steer it in the proper direction given the dearth of medical data and the myriad quality issues that accompany it. To maximize the overall efficiency of the systems, credible content that might be integrated into the deep architecture should be retrieved, for instance, from online medical encyclopedias and PubMed abstracts.
- Temporal Modeling: Training a swift DL model is crucial for improving understanding of the patient's state and providing prompt clinical decision support, since time plays a major role in many healthcare-related issues, especially those requiring monitoring devices and EHRs. For this reason, the resolution of healthcare problems requires temporal DL.
- Interpretable Modeling: Interpretability and model performance are critical when discussing health care issues. Medical practitioners are not likely to embrace a system that they do not completely comprehend. We believe that both ways of assisting the networks with currently available tools to explain the predictions of data-driven systems and algorithms for explaining the deep models—that is, the rationale behind the hidden units of the networks' decisions to turn on and off during the process—will be covered in future research directions.

### 6.3. Future aspects

#### 6.3.1. ML with AI

Future developments in ML and AI are anticipated to have a substantial impact on a variety of industries, including healthcare, finance, transportation, and many more. It is anticipated that AI will advance even further, eventually taking on activities that were previously thought to be solely humanly feasible. The following are some major factors that will probably influence how AI and ML develop in the future:

**Edge AI:** The increasing need for real-time AI processing and decision-making at the network edge will stimulate the rise of edge AI. Since data processing happens locally on the edge device, there's less need to transmit sensitive information to the cloud. This can enhance privacy and security, particularly in applications where data confidentiality is a priority of healthcare with low latency. Devices like wearable health monitors or medical imaging equipment can benefit from local AI processing. Real-time patient monitoring, remote diagnostics, predictive analytics for patient outcomes, emergency response systems, are the optimized results brought up with the help of edge AI.

**Explainable AI (XAI):** XAI refers to a set of strategies and methods designed to make AI systems' decisions and processes comprehensible and interpretable by humans. The lack of transparency in some AI models, particularly DL and complicated machine learning algorithms, can be a substantial impediment to their adoption in essential applications requiring trust and responsibility. Explainable AI seeks to overcome this issue by revealing how AI systems make certain decisions or predictions. Some common strategies used in XAI are explanation through features, local interpretable model-agnostic explanations (LIME), shapley additive explanations (SHAP), decision trees, and rule-based models. As AI systems become more intricate and pervasive, there will be a greater demand for the openness and explicability of their decision-making processes.

**DL on Graphs:** This is a brand new field of study that concentrates on using graph-structured data for DL models.

**RL:** RL is anticipated to become more crucial in AI, particularly in fields like robotics and autonomous systems. Overall, ML and AI have a bright future and are expected to make significant advances in a wide range of industries and applications.

#### 6.3.2. XAI with healthcare

The healthcare sector may undergo a change thanks to XAI, which increases the openness, accountability, and reliability of AI systems. Future uses for XAI include:

**Enhance diagnosis and treatment strategies:** By explaining their predictions and suggestions, XAI algorithms can assist medical professionals in making judgments that are more accurate and well-informed.

**Increase patient participation and training:** By using XAI, patients can receive more individualized information about their health and available treatments.

**Simplify clinical studies and research:** XAI can aid in the discovery of trends and connections in immense quantities of clinical evidence, facilitating the discovery of novel therapeutic approaches and treatments. Moreover, XAI may boost trust in AI systems by creating them to be more accessible and understandable, which would encourage healthcare practitioners to use them with more assurance.

#### 6.3.3. DL with 6G in cyber-physical system

The efficiency and effectiveness of cyber-physical systems (CPS) could be significantly increased by integrating DL and 6G. Future applications of this pairing include:

**Strengthen monitoring and efficiency:** In order to improve governance and increase the effectiveness of CPSs, DL algorithms may be used to examine the enormous volumes of data that these systems produce.

**Boost safety and dependability:** It will be feasible to improve security and reliability by collecting and analyzing data in instantaneously utilizing 6G's high speed, low latency, and high throughput capabilities. This will enable quicker and more informed decisions [230].

**Boost cybersecurity:** DL may be used to recognize and stop potential safety risks, and 6G's encrypted communication features can assist in assuring the privacy and protection of sensitive files. Again, integrating DL and 6G in CPSs can greatly enhance their capabilities and bring new levels of efficiency and safety to various industries.

#### 6.3.4. Federated learning with 6G in healthcare

The usage and sharing of healthcare data has the potential to be completely changed by the merging of federated learning and 6G. Future applications of this mixture encompass:

**Enhance customized drug:** By analyzing patient data from many sources using federated learning algorithms, more individualized treatment plans based on a wider variety of data are possible.

**Improved data privacy:** This is possible because of 6G's strong encryption features, which may help make sure that private patient information is secured when it is transferred amongst healthcare professionals.

**Streamline clinical trials and drug discovery:** Federated learning can aid in the discovery of trends and connections in vast volumes of medical studies, making it simpler to locate potential new remedies and cures.

**Facilitate telecare:** The deployment of telehealth apps that can bring direct remote consultations between healthcare practitioners and patients can be supported by 6G's high bandwidth and low delay.

#### 6.3.5. XAI with 6G in healthcare applications

The openness, accountability, and trustworthiness of AI systems in the healthcare industry might be significantly increased with the integration of Explainable XAI with 6G. Future applications of this pair tend to involve:

**Improve outcome:** XAI programs can offer more clear and explicable suggestions and forecasts to healthcare professionals, assisting in increasing the precision and knowledge of their choice.

**Enhance patient participation and skills training:** 6G's high speed and low latency can support the deployment of telemedicine petitions that can enable real-time remote counseling sessions between healthcare providers and patients. XAI can be used to educate patients while also providing them with more personalized information regarding their treatment and care alternatives.

**Aid to the invention or development of new Medicine:** XAI and 6G may be used to evaluate enormous volumes of clinical data, making it simpler to uncover potential new treatments and cures while still guaranteeing the confidentiality and safety of critical material. This will streamline clinical trials and drug development.

#### 6.3.6. 6G-based security in smart healthcare

The integration of 6G-based security in intelligent healthcare has the potential to significantly enhance patient data protection and confidentiality. Potential developments of 6G's security features usually involve:

**Privacy of Data:** Improved data privacy is possible because of 6G's secure communication and data storage capabilities, which may help guarantee that private medical information is safeguarded and kept private.

**Boost networked medical equipment' confidentiality:** 6G's encrypted communication capabilities can assist in preventing hacking and unauthorized access to medical devices, ensuring their safe and trustworthy functioning.

**Reliable Telehealthcare:** Facilitate the implementation of secure telemedicine apps that can enable real-time virtual consultations between medical physicians and patients while simultaneously protecting the privacy and security of sensitive data. 6G's rapid adoption and reduced latency can facilitate this.

**Safeguard Clinical Trials:** 6G's secure messaging capabilities may help ensure the confidentiality and preservation of sensitive trial information, making it simpler to carry out big clinical studies and create novel treatments and medications.

## 7. Conclusion

In this article, we conducted three different fields, including ML, DL and healthcare. This study thoroughly mines a massive amount of information based on tremendous areas such as ML, DL, and smart healthcare. Most significantly, we go into great depth on how ML and DL methods are now being used in smart healthcare applications. We presented vital features of ML, DL, and healthcare in a state of the art manner and significantly incorporated among them as ML-DL, ML-Healthcare, DL-Healthcare, and ML-DL-Healthcare. Notably, we focused on applications, challenges, and future opportunities efficiently. Though we have provided both ML and DL processes for the smart healthcare industry and classified them into various solutions, healthcare is wider than just ML and DL areas. Still, numerous issues and challenges need to be handled adequately. In the future, we plan to include security, privacy, and relevant issues with special attention. In addition, we provide further prospective study directions in this area.

## Use of AI tools declaration

The authors declare they have not used Artificial Intelligence (AI) tools in the creation of this article.
